# Observation and cholangioscopic biopsy of resectable gallbladder cancer using a new ultra-slim cholangioscope

**DOI:** 10.1055/a-2731-6152

**Published:** 2025-11-10

**Authors:** Tatsuya Kurokawa, Hirotsugu Maruyama, Yoshinori Shimamoto, Yuki Ishikawa-Kakiya, Kojiro Tanoue, Masanori Shiohara, Yasuhiro Fujiwara

**Affiliations:** 1 12935 Department of Gastroenterology, Graduate School of Medicine, Osaka Metropolitan University, Osaka, Japan; 2 12935 Department of Pathology, Graduate School of Medicine, Osaka Metropolitan University, Osaka, Japan

Endoscopic ultrasound (EUS)-guided tissue acquisition and percutaneous biopsy are not recommended for the preoperative diagnosis of resectable gallbladder cancer because of the risk of dissemination 1 . Definitive diagnosis using imaging tests is difficult, and transpapillary fluoroscopic biopsy and cytology lack high accuracy 2 . Direct observation and biopsy are desirable for a more accurate diagnosis; however, advancing a cholangioscope into the gallbladder through the narrow cystic duct is impractical. We report a case of successful direct observation and biopsy of a gallbladder cancer using a new ultraslim cholangioscope.

An 83-year-old woman was referred for a suspected gallbladder tumor based on a computed tomography scan ( Fig. 1 ). EUS revealed irregular wall thickening of the gallbladder and cystic duct, with continuous mild wall thickening of the common bile duct ( Fig. 2 ). Cholangiography revealed irregular gallbladder tumors, but no irregularities or stenosis in the common bile duct. An ultraslim cholangioscope (DRES Slim Scope and CMOS Camera; Japan Lifeline. Co. Ltd, Tokyo, Japan) was easily inserted into the gallbladder beyond the stenotic cystic duct. A papillary, friable, and irregular mucosa was observed from the gallbladder to the upper part of the cystic duct, whereas the mucosa in the lower part of the cystic duct and common bile duct was intact. Cholangioscopic biopsy of the gallbladder mucosa performed using a tapered sheath device (ERCP Guide Sheath; Olympus, Tokyo, Japan) and slim biopsy forceps (SpyBite Max; Boston Scientific, Tokyo, Japan; Video 1 , Fig. 3 ) revealed adenocarcinoma ( Fig. 4 ). The preoperative diagnosis was gallbladder cancer without bile duct invasion. There were no adverse events associated with the endoscopic procedure. The patient underwent surgical treatment, and the final diagnosis was the gallbladder cancer pT2N0M0.

**Fig. 1:**
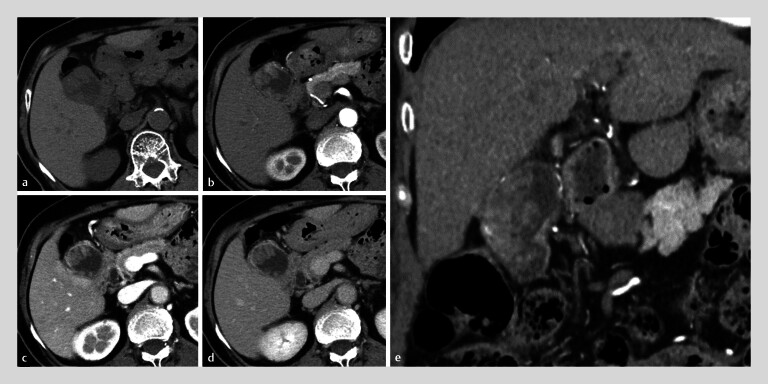
Computed tomography showing irregular wall thickening with enhancement in the gallbladder. **a** Axial, plane. **b** Axial, early phase. **c** Axial, portal phase. **d** Axial, delayed phase. **e** Coronal, early phase.

**Fig. 2:**
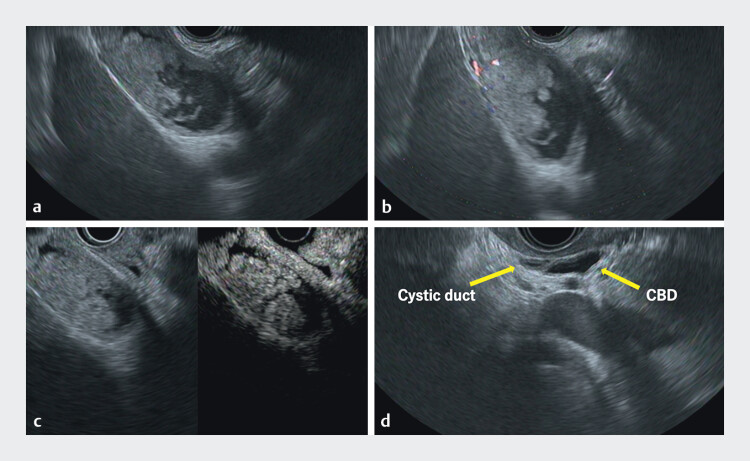
Endoscopic ultrasound images. **a** B mode imaging showing irregular wall thickening in the gallbladder. **b** and **c** Color doppler and contrast harmonic imaging confirmed blood flow within the lesion. **d** B mode imaging showing the cystic duct and common bile duct with mild wall thickening.

Cholangioscope showing a papillary, friable, irregular mucosa of the gallbladder. Observation and cholangioscopic biopsy of resectable gallbladder cancer using a new ultra-slim cholangioscope.Video 1

**Fig. 3:**
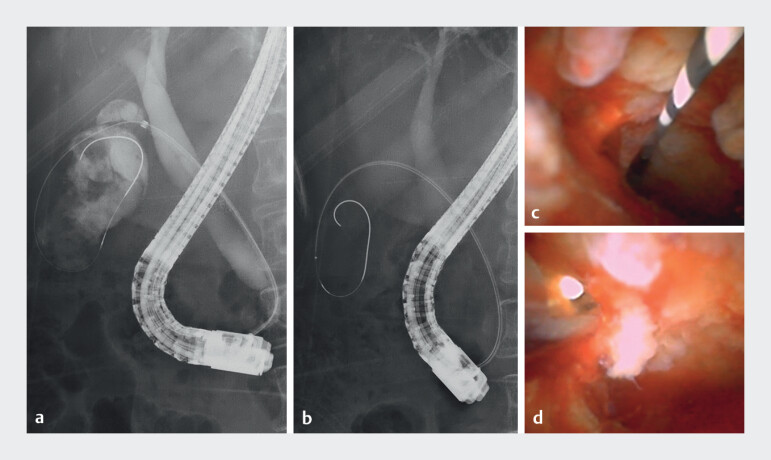
Cholangiography and cholangioscopy. **a** Cholangiography showing no irregularity or stenosis in the common bile duct and irregular gallbladder tumor. **b** An ultra-slim cholangoscope was inserted into the gallbladder. **c** Cholangioscope showing a papillary, friable, irregular mucosa of the gallbladder. **d** Cholanigoscopic biopsy of the gallbladder mucosa.

**Fig. 4:**
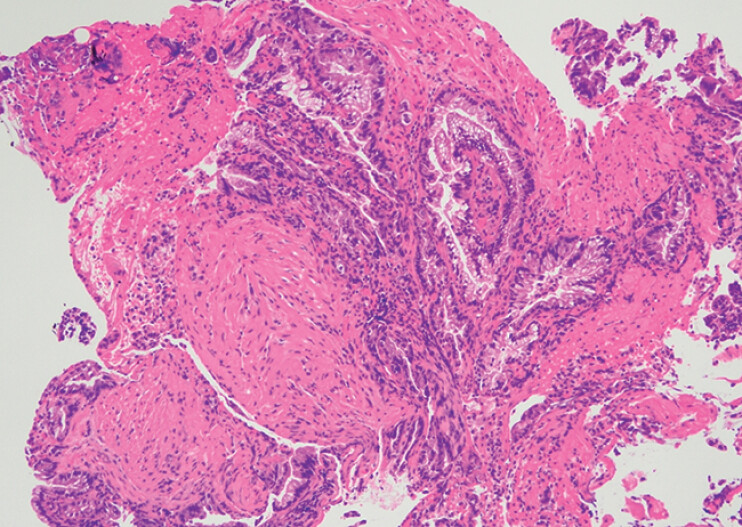
Biopsy specimen showing epithelium with marked atypia and diagnosed as adenocarcinoma.

There are few reports on cholangioscopic biopsy for gallbladder cancer; however, it may be a promising new diagnostic option for resectable gallbladder cancer 3 .

Endoscopy_UCTN_Code_TTT_1AR_2AD

## Notes

Endoscopy E-Videos https://eref.thieme.de/e-videos
*E-Videos* is an open access online section of the journal *Endoscopy* , reporting on interesting cases and new techniques in gastroenterological endoscopy. All papers include a high-quality video and are published with a Creative Commons CC-BY license. Endoscopy E-Videos qualify for HINARI discounts and waivers and eligibility is automatically checked during the submission process. We grant 100% waivers to articles whose corresponding authors are based in Group A countries and 50% waivers to those who are based in Group B countries as classified by Research4Life (see: https://www.research4life.org/access/eligibility/ ). This section has its own submission website at https://mc.manuscriptcentral.com/e-videos .
